# Real-Time Action Recognition System for Elderly People Using Stereo Depth Camera

**DOI:** 10.3390/s21175895

**Published:** 2021-09-01

**Authors:** Thi Thi Zin, Ye Htet, Yuya Akagi, Hiroki Tamura, Kazuhiro Kondo, Sanae Araki, Etsuo Chosa

**Affiliations:** 1 Graduate School of Engineering, University of Miyazaki, Miyazaki 889-2192, Japan; ietei.n0@cc.miyazaki-u.ac.jp (Y.H.); mjakagi@icloud.com (Y.A.); htamura@cc.miyazaki-u.ac.jp (H.T.); 2 Faculty of Medicine, University of Miyazaki, Miyazaki 889-1692, Japan; kondokaz@med.miyazaki-u.ac.jp (K.K.); saraki@med.miyazaki-u.ac.jp (S.A.); chosa@med.miyazaki-u.ac.jp (E.C.)

**Keywords:** ambient assisted living, stereo depth camera, UV-disparity maps, depth map features, depth motion appearance, depth motion history, histogram of oriented gradients, action recognition

## Abstract

Smart technologies are necessary for ambient assisted living (AAL) to help family members, caregivers, and health-care professionals in providing care for elderly people independently. Among these technologies, the current work is proposed as a computer vision-based solution that can monitor the elderly by recognizing actions using a stereo depth camera. In this work, we introduce a system that fuses together feature extraction methods from previous works in a novel combination of action recognition. Using depth frame sequences provided by the depth camera, the system localizes people by extracting different regions of interest (ROI) from UV-disparity maps. As for feature vectors, the spatial-temporal features of two action representation maps (depth motion appearance (DMA) and depth motion history (DMH) with a histogram of oriented gradients (HOG) descriptor) are used in combination with the distance-based features, and fused together with the automatic rounding method for action recognition of continuous long frame sequences. The experimental results are tested using random frame sequences from a dataset that was collected at an elder care center, demonstrating that the proposed system can detect various actions in real-time with reasonable recognition rates, regardless of the length of the image sequences.

## 1. Introduction

Because of recent advances in medicine, people live longer than before, partly accounting for a rapid increase in population. As one result, society is aging compared to that of previous generations. Society and health care systems face many demanding challenges because of this aging population. Health care costs are rising, and we have a shortage of well-trained caregivers. The longer people live, the more that senior citizens must be concerned with physical and mental health problems, which impact budgets for health care costs. Older adults cannot always live independently in their homes due to age-related diseases, as well as limitations in physical activity, vision, and hearing. Family members must care for them or hire professional caregivers at home or at care centers. Society must respond to these needs and solve these challenges in a timely manner. In recent years, researchers have developed assistive technologies, which can empower people in the digital environment. One such technology is called ambient assisted living (AAL).

Ambient assisted living (AAL) is a healthcare-monitoring system consisting of smart devices, medical sensors, wireless networks, computers, and software applications. AAL can be used for various purposes, such as curing diseases, improving health, and preventing difficulties for older adults [1]. AAL can help the elderly to live independently in an environment of their own preference. As a matter of fact, AAL can increase safety for the elderly using mobile emergency alert systems [2], fall detection systems [3,4,5], and video surveillance systems [6], as well as reduce the intrusive impact of caregivers on their lives.

Recent advances in several technological areas have helped the vision of AAL to become a reality. These technologies include smart homes, assistive robotics, as well as mobile and wearable sensors. Likewise, AAL systems for the elderly have been analyzed in recent years through research using wearable sensors, ambient sensors, and vision sensors. As for sensor-based methods, some researchers have proposed systems that use accelerometers as well as gyroscopic sensors on smartphones and smartwatches [7,8]. These systems include for motion-based biometric authentication and identification, based on an understanding of the subjects’ behavior obtained by tracking their daily routines, such as their eating habits. Furthermore, the Internet of Things (IoT) has gained much attention from researchers in recent years as it provides opportunities for developing smart healthcare systems by connecting a variety of medical sensors and devices with people. For example, among previous studies, a non-intrusive, spatial-temporal abnormal behavior detection system [9] was introduced for monitoring elderly people living alone. This system combines IoT sensors and machine learning techniques to provide a home-based solution. Some other researchers use ambient sensors (non-wearable sensors) for elderly care and behavioral monitoring. Most of these sensors are motion, radar, object pressure, and floor vibration sensors [10]. Systems featuring wearable sensors can be intrusive, being uncomfortable to wear all the time. On the other hand, ambient sensors can be expensive, requiring the installation of variety of sensors and controller hardware. Such problems can be tackled by using vision-based monitoring systems. Most users prefer such systems for their ease of installation, low cost, and preclusion of wearable sensors. Moreover, the field of computer vision has been advancing steadily over the years, attracting interest among researchers and in industry.

For vision-based methods, another system focused on a monitoring system designed to be as unobtrusive as possible [11]. This system features a home installation of visual sensors with RGB cameras, computer vision techniques, and action recognition using a machine learning approach. Although vision-based systems achieve robust accuracy rates for recognizing most human actions, disadvantages include privacy issues, especially if systems are set up in care centers or hospitals. Recent advances in depth cameras motivate their use in place of RGB cameras or wearable sensors as they provide advantages such as privacy protection. In addition, advances in 3D scene acquisition enable 3D modeling and body pose estimation in real time, doing so in solutions that are affordable and simple to set up. Such advances are relevant to the field of assisted living. To address privacy concerns, a secure cloud-based solution [12] is proposed for recognizing human actions using color and depth data. For this solution, researchers only collected motion history images [13] generated from color data and depth motion maps generated from depth data, using a deep convolutional neural network (CNN) to perform recognition.

In the proposed system, a stereo depth camera is used to recognize the various actions of the elderly at an elder care center. The objective of this work is to provide a generic solution for long-term, practical applications by preventing accidents and supporting the health of residents in care centers. To recognize the various actions of people using depth sensors, extracting useful features is very important. As another consideration, depth cameras operate on numerous categories of data representation [14], such as skeletal and depth maps, point clouds, and plan-view maps. Among these, skeletal and depth maps are the most useful for human action recognition. For example, some previous works [15,16] propose recognizing daily activities of the elderly using depth-based systems that obtain skeletal joint information from the depth silhouettes of human. Furthermore, a recognition system [17] was also introduced that only uses depth motion maps of human silhouettes obtained from three perspectives (front, side, and top). These silhouettes are used to capture the motion characteristics of an action sequence. Sometimes two representations (skeletal and depth maps) are fused together to improve recognition accuracy. Recently, an action recognition system was proposed [18] using a decision-level fusion of both skeletal and depth map sequences. Moreover, transformations of depth maps to point clouds in classifying human activities were emphasized by some studies [19,20].

As a disadvantage of using skeleton joint information from depth cameras, extracting joint information is only suitable for a person facing the camera, and can fail when the person is not in an upright and frontal view position such as when “Lying on the bed”. The camera can also provide false joint information when occlusion occurs. As limitations of using point-cloud features, the approach involves high computational complexity when converting from depth maps to a point cloud, and presents difficulties in extracting point cloud features for action recognition. To address these limitations and drawbacks, the proposed system uses a stereo depth camera that only provides depth sequences, thus focusing on a way of detecting, tracking, and recognizing actions using depth maps exclusively. From these depth maps, a person’s silhouette can be easily extracted. In addition, depth maps provide body shape information for the whole side facing the camera [3], rather than just the 2D information provided by the silhouette.

Many algorithms have been proposed to recognize actions using representations built from 3D silhouettes [6,21], as well as using depth motion maps [22] obtained from 3D silhouettes. Among these, a threshold-based action recognition algorithm using distance features from 3D silhouettes [21] was recently proposed. In this system, the 3D human centroid height relative to the floor is used as a feature for action recognition, and the percentage of body movement from frame differencing is used for sleep monitoring. Then, pre-defined threshold values are used for action recognition rather than classification techniques. After that, the system recognizes situations such as the person location (Inside and Outside), specific actions (Sitting and Lying), and sleep behaviors (Sleeping well, Minor body movements, Major body movements). However, this system is very sensitive to small changes and can make false detections, as when the person is occluded by another person, or objects such as blankets, curtains, and wheelchairs. These false detections occur because the recognition decision is made by only using the centroid point of the person’s silhouette. Furthermore, this method is not useful for any indoor environment at elder care centers, as the depth images provided by the proposed depth camera have large depth noise variations, and thus, the pre-defined threshold values will differ from one indoor environment to another. To address this issue, this previous action recognition system [21] is extended in our proposed system by extracting the spatial-temporal features from the depth maps which are fused together with distance features to improve accuracy and reliability.

There are some previous works which relied on spatial-temporal features for action recognition. For instance, a spatial and temporal attention model [23] was designed to explore discriminative features for human action recognition and detection using skeleton data. In addition, some researchers extracted depth motion and appearance features in multiple views using depth maps, obtaining feature descriptors from the histogram of oriented gradients (HOG) for each depth map [24]. The proposed work adopts ideas from the previous work [24], but unlike the previous work, the spatial-temporal features are only extracted for a single front view from the camera. This greatly reduces the computational complexity of the feature extraction process.

In the proposed system, data are collected throughout the day inside the bedrooms of the elder care center over durations of one to two weeks. The acquired dataset thus contains long, continuous frame sequences. Our objective also is to create a real-time monitoring system that continuously processes input frames. Four strategies are usually applied to handle long frame sequences: manual annotation [25], pre-temporal video segmentation [26], sliding windows [27], and automatic rounding methods [28]. Moreover, a novel action-recognition framework was recently proposed for use with long, continuous video. This framework is called long-term video action recognition [29]. For the sake of simplicity, our experimental approach features the automatic rounding method [28] to recognize various actions in long sequences. In the previous rounding method [28], the optical flow was used as a low-level measure of activities in local regions for the feature extraction of each frame. Then, the model was fitted with the expectation maximization (EM) algorithm in the training stage, and a Bayesian decision was applied in the testing stage. Next, it automatically divides long frame sequences into several short rounds and decides whether an action has occurred or not. However, this recognizer would sometimes provide an incorrect result if a recognition round has not stopped before a new action starts. For this issue, we set a pre-defined maximum duration. If due to low probability, the recognition round does not stop until reaching a maximum duration, the recognizer considers the sequence to be an undetermined action. Otherwise, the recognizer labels a specific action. In contrast with the previous rounding method, our system fuses spatial-temporal features and distance features, and uses an SVM classifier for decision making. Moreover, most of the previous action recognition systems were tested using publicly available datasets, which were annotated for specific actions of short duration by ignoring the issue of action recognition for continuous long video sequence in real time. By contrast, our experiment records and then tests continuous long real-world data.

Residents of the care center stay outside their rooms during most of the day. They usually enter the room in their wheelchairs (all participants in this experiment used wheelchairs), stand up from the wheelchair, sit on the bed, and finally lie down to rest (they usually take a nap for 2 to 3 h in the afternoon, and sleep for 10 to 12 h at night). They perform these actions by themselves or assisted by a nurse. Accidents such as falling could occur during transitions from one action to another, which poses serious danger for the elderly.

Given the above-mentioned daily routines, we intend to recognize actions which the residents perform inside their rooms at the care center when no nurses are present. We recognize the following actions in this experiment: “Outside the room”, “Transition”, “Seated in the wheelchair”, “Standing”, “Sitting on the bed”, “Lying on the bed”, “Receiving assistance”, and “Falling”. We firstly determine whether the residents are inside or outside the room. If inside, we further recognize the other actions. Actions such as eating, walking, and exercising are usually performed outside their rooms. However, the participants in this experiment could not walk or exercise on their own. Furthermore, this experiment does not involve complex actions such as changing clothes, taking medicine, or folding blankets.

Because of the risk of falling, recognizing transitions is important in this experiment. The risk is greatest during transitions between sitting and standing, and between sitting and lying down. If the elderly person makes too many transitions between sitting and standing, we also know that this person is having difficulties during these transitions. As part of understanding these transitional states, we must recognize the specific actions leading up to these transitions. Next to falling, recognizing these actions is critical to ensuring the well-being of the elderly. For example, if the duration of the elderly person lies on the bed is more than normal, we can conclude that this person faces some sleep-related problems.

By using this system to recognize the actions of the care-center residents, we can not only monitor and assess their health, but also record their action histories and behaviors. Automated analysis of these recordings further ensures the safety of residents, preventing difficulties, and allowing timely diagnosis and treatment of maladies. This can also greatly reduce the workload of caregivers as an AAL system.

The rest of this article is organized as follows. Section 2 presents the used methodology by introducing the data acquisition method, providing a system overview, and describing the details of each method used in the system. Section 3 explains the experimental results, and quantitatively evaluates the performance of the action recognition process. Finally, Section 4 provides discussion and concluding remarks.

## 2. Used Methodology

This section concerns the data acquisition method using stereo depth cameras and provides a process overview for the proposed system.

### 2.1. Data Acquisition

**Figure 1:**
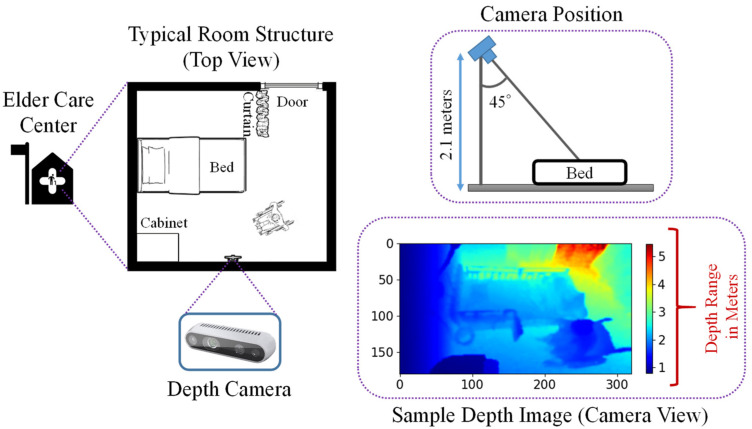
A typical room inside the elder care center, also showing the camera position, and a sample depth image.

The depth cameras were set up for data acquisition inside the rooms of an elder care center in Miyazaki Prefecture, Japan. A top view of a typical room is shown in Figure 1. Three elderly people participated in this experiment in three separate rooms, with their daily activities inside the room recorded using a stereo depth camera. This camera was placed 2.1 m above from the floor, viewing the room at a downward angle. A stereo depth camera only provides data for the distance between camera and objects, and we collected these data in a comma separated values (CSV) file format, which we then converted to image data. The image resolution was 320 × 180, recorded at a rate of 1 fps (frame per second). Figure 1 also provides a sample depth frame for an elderly person seated in a wheelchair, with a color-coded map representing the range of distances in meters from the camera to the objects. Blue pixels represent objects near the camera, while red pixels represent those farther away. The RGB frames were not recorded in this experiment due to privacy issues.

### 2.2. System Overview

**Figure 2:**
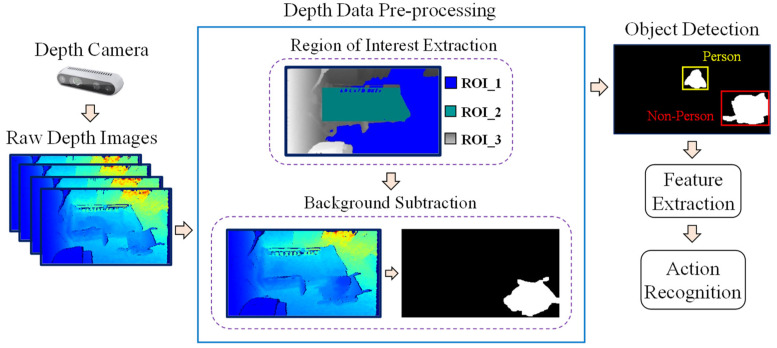
Overview of the proposed system.

An overview of the proposed system is shown in Figure 2. The depth camera first generates raw depth images. These depth data are then pre-processed using region of interest (ROI) extraction and background subtraction methods. After that, object detection and tracking are performed, and different features are extracted from the detected person’s silhouettes. These features are used as input to the classifier, and then the various actions are recognized. The details of each process will be described in subsequent sections.

### 2.3. Depth Data Pre-Processing

The data recorded by the depth camera are raw distance data which contain various types of noise. For instance, some depth images contain many holes which lack depth information due to camera error, and some images include much depth-variation noise. Therefore, these data must be pre-processed to facilitate manipulation in subsequent steps. For this purpose, each depth frame is enhanced by filling holes and filtering. Holes are filled with black pixels where information has been lost. Filtering is used for smoothing images and reducing noise. This experiment incorporated the “filling-from-left” method for filling holes, and the “bilateral” method for filtering, which can preserve edges.

**Figure 3:**
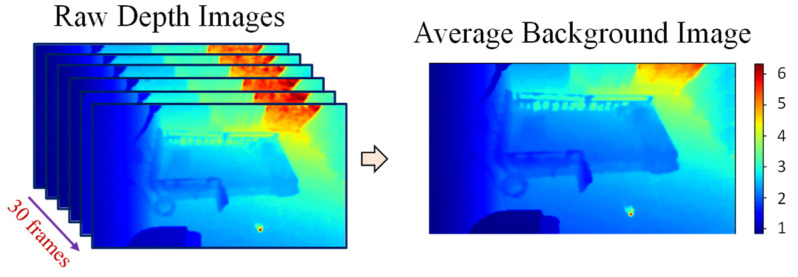
Average background image.

After that, the average of the first 30 pre-processed frames not including people is calculated and used as the background image, as shown in Figure 3. This average background image will be used for background subtraction in the next step. Furthermore, some pixels that are distant from the camera are also removed from the average image to reduce unstable depth noise. Finally, various ROIs are extracted from the image.

#### 2.3.1. ROI Extraction

First, locating the person in the room facilitates the image processing involved in recognizing actions. For this reason, different regions of interest within the camera view are extracted from the background image for person localization. After background subtraction, additional noise can be removed from unwanted areas once identifying the regions in which pixels are located. ROI extraction here involves the use of the depth-disparity relationship and the V-disparity map. Three regions are extracted in this experiment, namely, the floor region, bed region, and ignored region.

**Figure 4:**
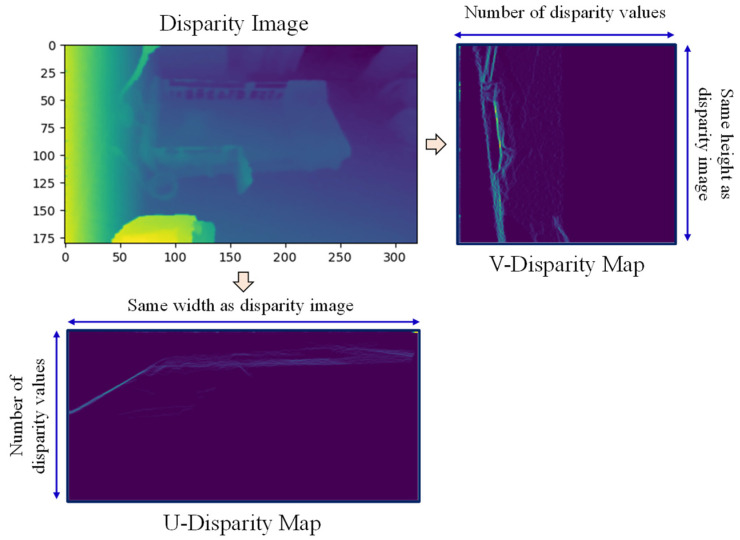
Disparity map to UV-disparity map conversion.

- Disparity map:
  The images from the depth camera represent actual distances (depth), and this depth data can be converted to a disparity map for extracting the regions. Formally, the disparity values are inversely proportional to the depth values, and the conversion of a depth map to a disparity map is described in Equation (1). A disparity map can also be defined as the apparent pixel difference between a pair of stereo images as in Equation (2), where x and $x^{\prime }$ represent pixels from left and right images, respectively.
  (1) $$\mathrm{Disparity}\;\left(\mathrm{pixels}\right)\;=\;\frac{\mathrm{Focal}\;\;\mathrm{Length}\;\left(\mathrm{pixels}\right)\times \mathrm{Baseline}\;\left(m\right)}{\mathrm{Depth}\;\left(m\right)}$$
  (2) $$\mathrm{Disparity}=x-x^{\prime }$$
- UV-disparity maps:
  After the depth-to-disparity conversion, the disparity map can also be transformed into two other disparity maps named the U-disparity and V-disparity maps [30,31]. An example of a conversion from a disparity map to a UV-disparity map for the average background image is shown in Figure 4. The V-disparity map is the histogram image of disparity values for each row of the depth image. As such, it has the same height as the disparity image, and the width equals the number of disparity values. Similarly, the U-disparity map is the histogram image of disparity values for each column of the depth image, having the same width as the disparity image, with a height that equals the number of disparity values. By converting from a disparity map to a UV-disparity map, the flat surfaces in both horizontal and vertical directions can be extracted.

**Figure 5:**
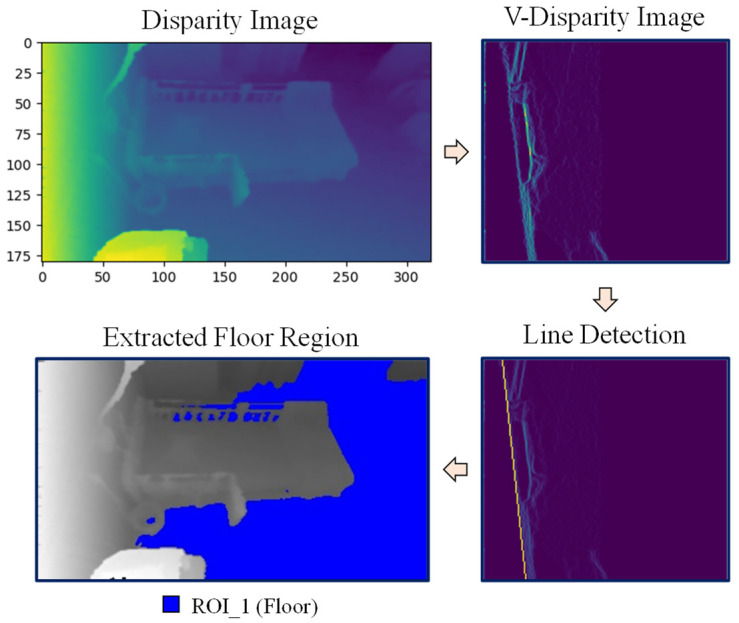
Floor region extraction.

The following describes the process of floor region and bed region extraction using the above conversions. For extracting the floor region, the V-disparity map is used, and this process is shown in Figure 5. In the V-disparity map, the floor plane is usually simplified into a slanted straight line which is called the ground correlation line. Basically, the disparity values increase linearly along the floor plane, and the Hough line transform is used to detect this line. With this V-disparity map and the straight line corresponding to the floor plane, the pixel indices which belong to the straight line can be detected as floor pixels.

**Figure 6:**
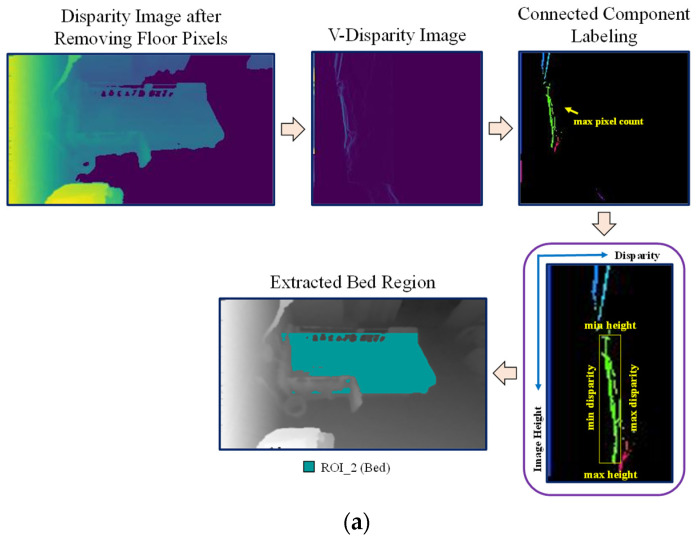

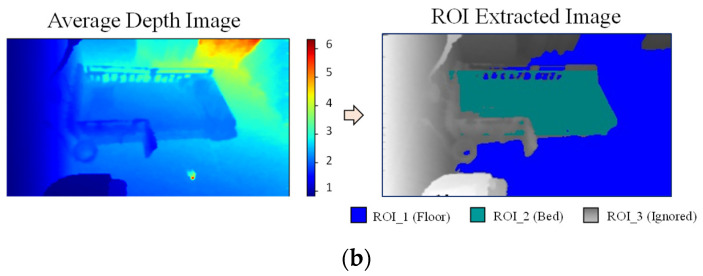
(**a**) Bed region extraction; (**b**) Three ROI regions.

After removing floor pixels, the disparity map without floor pixel values is used to extract the bed region, as shown in Figure 6a. The V-disparity map is also used for extracting the bed region, but we will not find a straight line in this approach as the bed surface is not as flat as the floor. Instead, connected component labeling is used to find the label with the largest pixel count in the V-disparity map. The assumption is that the bed has the largest area as compared to other objects in the bedroom. After that, four extreme points (minimum height, maximum height, minimum disparity, and maximum disparity) are extracted from the detected label which has the maximum pixel count. We used these four points to establish the bed pixels. From the minimum to maximum height in the disparity image, the pixels which have disparity values within minimum and maximum disparities are detected as bed pixels. Finally, the detected floor pixels and bed pixels are subtracted from the average background depth image. The remaining pixels are automatically detected as the ignored region as shown in the Figure 6b, in which the three ROI regions are represented by three different colors.

**Figure 7:**
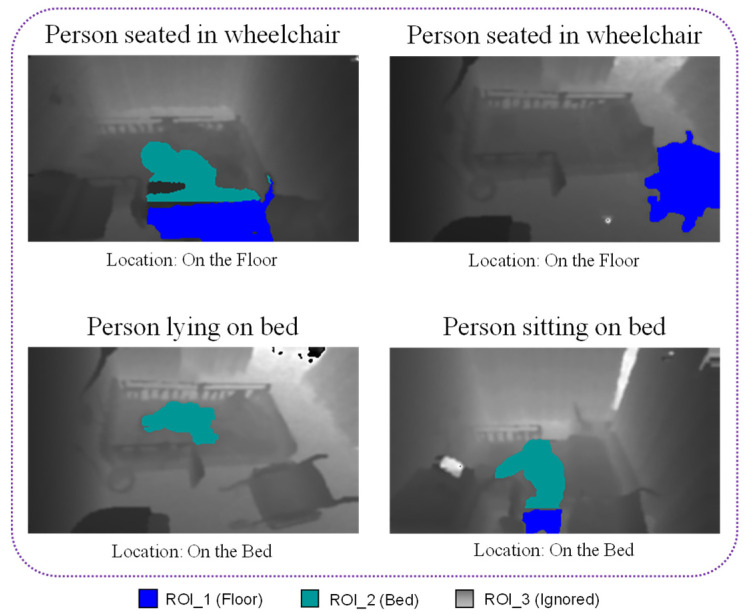
Person localization for different frames.

After ROI extraction, the pixels belonging to different regions are stored, and pixel intersection is used to locate the person in the room. For instance, if more than 70% of pixels in a blob recognized as a person are located in the bed region, it can be deduced that the person is located on the bed. However, if more than 30% of the blob is located in the floor region, that person is located on the floor. An example of person localization for different frames is shown in Figure 7, in which the color representations for each region are also visualized.

#### 2.3.2. Background Subtraction

**Figure 8:**
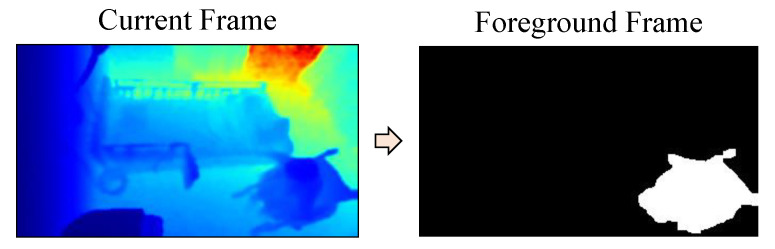
Background subtraction.

The background image is subtracted for clearly extracting objects in the foreground. In this experiment, simple static frame differencing is used for background subtraction. To get the foreground image *F*, the current depth image *I* is subtracted from the average background image *B*, and compared with the threshold *T* for each pixel (*i*,*j*), as shown in Equation (3). The threshold value is based on the standard deviation of the current depth image. An example of background subtraction for the frame of an elderly person seated in a wheelchair is shown in the Figure 8.

(3) $$F(i,j)=\left\{\begin{array}{ll} \;\;1 & \mathrm{if}\;\left|B(i,j)-I(i,j)\right|\geq T(i,j),\\ \;\;0 & \mathrm{otherwise}. \end{array}\right.$$

#### 2.3.3. Curtain Removal

**Figure 9:**
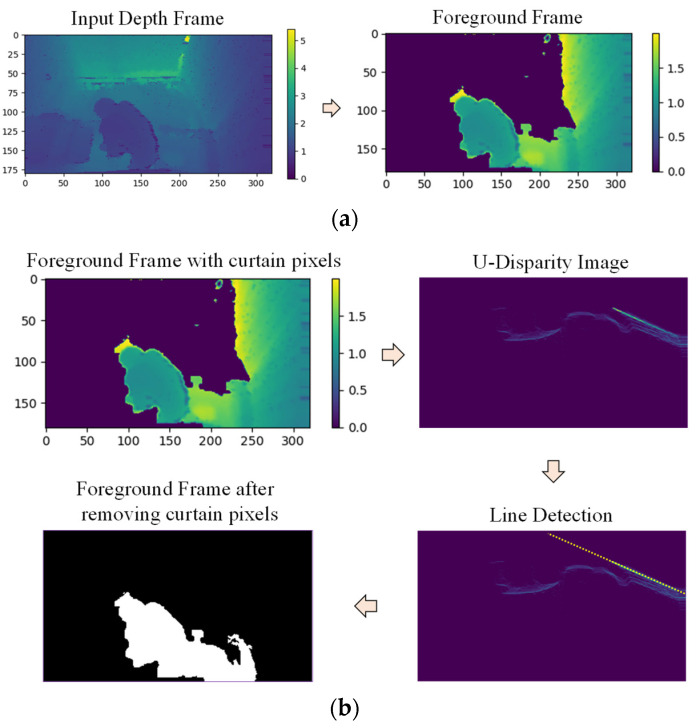
(**a**) Example of foreground frame with curtain; (**b**) Process of curtain removal.

The foreground objects are extracted after background subtraction, but some frames contained unwanted noise. Some noise can be eliminated using morphological operations. However, the view of each room is obscured by a curtain at night, and these curtains appear as noise in some frames. An example of a foreground frame with curtain pixels is shown in Figure 9a. In this example of a depth frame, an elderly person is using a bedside commode at night, and the view is obscured by a curtain. As these curtain pixels complicate action recognition, they must be removed as part of handling foreground objects. The process of curtain removal is also shown in Figure 9b.

Curtain pixels can be detected using the U-disparity map because the structure of the curtain is flat and the disparity values increase along the flat surface of the curtain in the U-disparity map. The Hough line transform is used to detect a straight line in the U-disparity map, which indicates the presence of a curtain. The pixels belonging to the straight line are then identified as curtain pixels and removed.

### 2.4. Object Detection and Tracking

After removing noise and curtain pixels from the foreground objects, the person will be detected from the remaining objects. The person is detected with the assumption that the person is moving for some duration while non-person objects are not moving at all. According to this assumption, motion information is used to detect the person.

**Figure 10:**
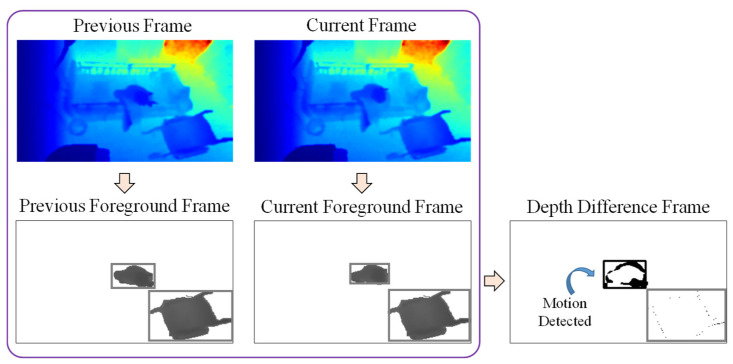
Example of motion detection for an elderly person sitting on the bed.

Firstly, the depth difference between two consecutive frames is calculated using Equation (4) where D(i,j) denotes the depth value at position (*i*,*j*), and *t* and *t −* 1 denote the frame indices. An example of motion detection is shown in Figure 10, in which the upper bounding box of each foreground frame contains the elderly person sitting on the bed, and the lower bounding box contains the wheelchair. The sum of this depth difference within the bounding box of the current frame is then calculated as in Equation (5), where $B_{i}$ is the bounding box at the *i*th foreground object of the current frame. If the condition $\Delta D(B_{i})>Th_{motion}$ is satisfied, motion has been detected in this bounding box. In this example, the upper bounding box is identified as a moving object because the elderly person is moving while sitting on the bed, and the black pixels represent the depth differences. Thus, this object can be represented as a person object by deciding within two frames, which is a duration of two seconds. However, the wheelchair object in the lower bounding box might also be identified as a person if another object passes through the wheelchair object, increasing the depth difference in this bounding box. Because of this, we wait 10 frames (10 s) to be sure that the object is a person. In this experiment, if motion is detected for more than six seconds within a duration of 10 s, a final decision is made that the foreground object is a person.

(4) $$\delta D(i,j)=D_{t}(i,j)-D_{t-1}(i,j)$$

(5) $$\Delta D(B_{i})=\sum_{(i,j)\in B_{i}}\delta D(i,j)$$

To determine the history of movement for an object identified as a person, and also to track the object after person detection, a simple association rule is used. This association rule is applied for two consecutive frames. For this association, the Euclidean distance between two centroids $E\_Dist(C_{t-1},C_{t})<Th_{dist}$, the blob area difference $\left|A_{t-1}-A_{t}\right|<Th_{area}$, and the average depth difference $\left|D_{t-1}-D_{t}\right|<Th_{depth}$ of detected objects are used, where *C* is the centroid, *A* is the blob area, and *D* is the average depth value, respectively. If the conditions of the detected objects in two consecutive frames are associated for all of these constraints with pre-defined thresholds, they can be identified as the same objects.

### 2.5. Feature Extraction

Regarding feature extraction, the depth camera we used only provides depth-map sequences, and does not provide any additional information such as the joint features provided by Kinect depth sensors (Microsoft, Washington, DC, USA). Thus, the experiment focused on the extraction of important features from the depth maps.

#### 2.5.1. Appearance-Based Features

**Figure 11:**
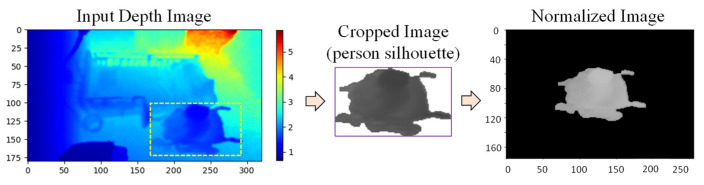
Normalization of person silhouette.

**Figure 12:**
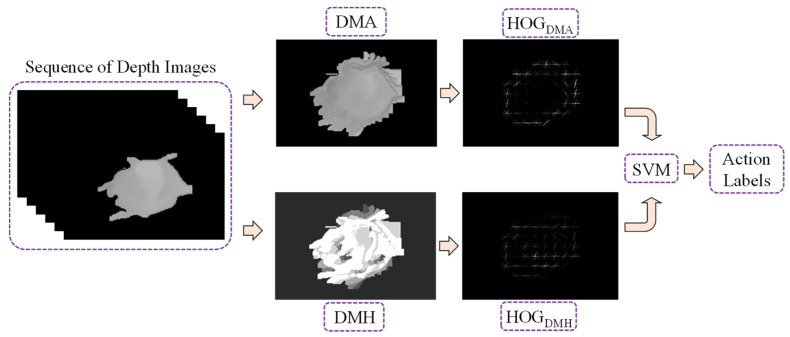
Feature extraction and classification framework.

In this experiment, space–time features from a sequence of depth silhouettes for a person are used for feature extraction, because we can obtain little information from the depth map if we extract features from only one frame. For an entire action sequence, action representation maps called depth motion appearance (DMA) and depth motion history (DMH) are extracted from the sequence of depth maps. Then, the histogram of oriented gradients (HOG) is used as a feature descriptor for each map, which can be described as a more relevant and insightful representation. In order to calculate the two maps, the person silhouettes are first cropped from the depth map, and then normalized to a fixed size by centering the silhouettes with zero padding, as shown in Figure 11. A framework for feature extraction and classification is shown in Figure 12, using the depth frame sequences of an elderly person seated in a wheelchair as an example.

- Depth motion appearance (DMA):
  Firstly, the DMA is a volumetric representation of the overall shape and appearance of the depth motion obtained by organizing and combining all of the depth images in order throughout the action sequence [24]. As a matter of fact, the DMA uses 3D information for the entire depth map in the sequences. The DMA can be calculated using Equation (6), where $D_{t}(i,j)$ is a depth value at pixel location (*i*, *j*) of the *t*th input depth map, and $DMA_{t}(i,j)$ is a depth value at pixel location (*i*, *j*) of the DMA generated from the *t*th input depth maps. With the DMA, the appearance and global 3D shape can be extracted for the motion within a specific duration.
  (6) $$DMA_{t}(i,j)=\left\{\begin{array}{cc} \;D_{t}(i,j) & \text{if}\;\;DMA_{t-1}(i,j)=0,\\ \;min(D_{t}(i,j),DMA_{t-1}(i,j)) & \text{otherwise}. \end{array}\right.$$
- Depth motion history (DMH):
  As for the actual action performed in the real world, we identify the action using common sense by not only determining the appearance history, but also the temporal history of a motion. Although the DMA can provide the appearance of motion forming an action, it cannot provide temporal information throughout the sequence. For this purpose, the DMH is also used to extract dynamic temporal features in this experiment. The DMH is an expansion map of the motion history image (MHI) used for extracting dynamic information. The MHI is typically used on 2D images, and the depth information is added to this algorithm for calculating the DMH from these depth images. By accumulating this depth information, changes in direction detected in past body movements can also be concealed [24]. The DMH can be calculated from Equation (7), where $DMH_{t}(i,j)$ denotes a historical value for depth motion at pixel location (*i*, *j*) of the DMH generated from the *t*th input depth maps, *τ* is a time window for that history, and *δ* is a threshold value for the depth difference between consecutive depth maps.
  (7) $$DMH_{t}(i,j)=\left\{\begin{array}{cc} \;\tau & \text{if}\;\;\left|D_{t}(i,j)-D_{t-1}(i,j)\right|>\delta ,\\ \;max(DMH_{t-1}(i,j)-1,0) & \text{otherwise}. \end{array}\right.$$
- Histogram of oriented gradients (HOG):
  After gathering the two action representation maps, the HOG is employed as a feature descriptor to describe the local appearance and shape of the DMA and DMH. The HOG approach evaluates the existence of gradient orientation in the localized and confined parts of an image. Person silhouettes are cropped from the depth map of the sequence, and then normalized to a fixed size of 256 × 176 with zero padding, before calculating the two maps. Each map is divided into 16 × 16 pixels per cell, the HOG is calculated for each cell with nine gradient directions, and the L2-norm is then used for block normalization. The histogram is then normalized using a 1 × 1 block for each cell, with 16 horizontals and 11 vertical positions. Each feature map becomes a HOG descriptor with dimensions of 16 × 11 × 9 = 1584. Finally, the two histograms from two maps are concatenated and a 1584 × 2 = 3168 dimensional HOG descriptor is obtained for the entire action sequence. After that, this fusion histogram is used to train a multi-class linear support vector machine (LinearSVM), exploiting classifications for the various action labels.

#### 2.5.2. Distance-Based Feature

**Figure 13:**
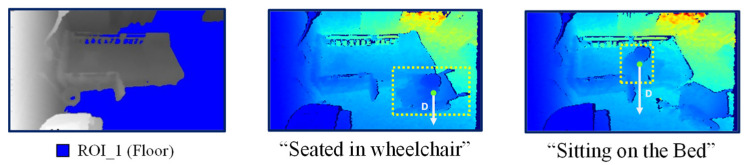
Examples of distance feature extraction.

Additionally, the distance feature from the centroid of the person to the floor plane is also extracted to determine the person’s height. The process of distance feature extraction is shown in Figure 13. Extracting this feature first involves using Equation (8) to construct the floor plane equation from the already extracted floor pixels (*x_f_,y_f_*) along with their corresponding depth values *z_f_*, and then calculating parameters *a*, *b*, *c*, and *d* using the least squares method. When a person enters the room and a person silhouette is detected, the 2D centroid point for this silhouette is extracted. This point is defined as (*x*,*y*) in pixel coordinates, which are converted to the 3D points (*X*,*Y*,*Z*) in camera coordinates using Equation (9), where (*c_x_,c_y_*) is the center of image and *f* is the focal length in pixels. Finally, the distance *D* from the centroid to the floor plane can be calculated using point-plane equation in Equation (10).

(8) $$ax_{f}+by_{f}+cz_{f}+d=0$$

(9) $$X=\frac{Z(x-c_{x})}{f},\;Y=\frac{Z(y-c_{y})}{f}$$

(10) $$D=\frac{\left|aX+bY+cZ+d\right|}{\sqrt{a^{2}+b^{2}+c^{2}}}$$

### 2.6. Action Recognition

**Figure 14:**
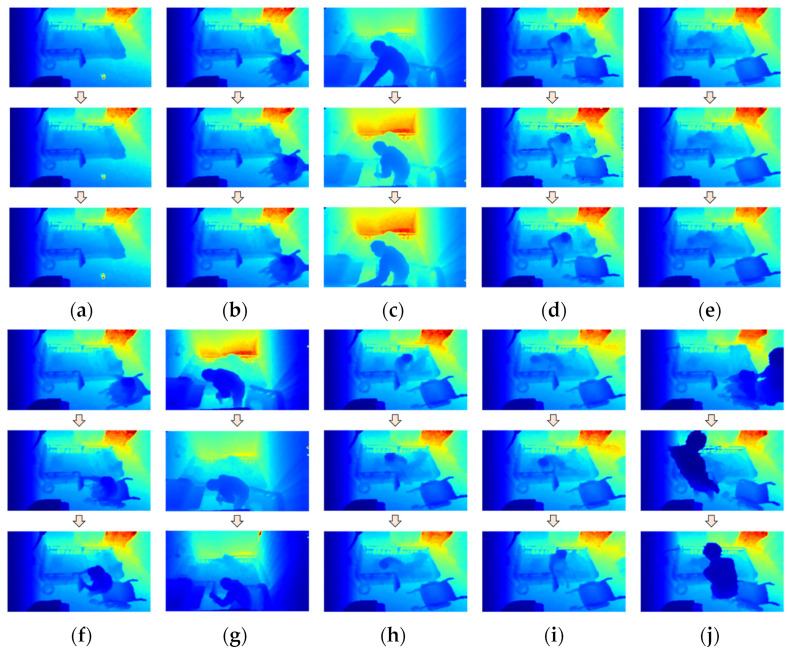
Example depth sequences for action labels: (**a**) Outside the room; (**b**) Seated in wheelchair; (**c**) Standing; (**d**) Sitting on bed; (**e**) Lying on bed; (**f**) Transition (sitting to standing); (**g**) Transition (standing to sitting); (**h**) Transition (sitting to lying down); (**i**) Transition (lying down to sitting); (**j**) Receiving assistance.

Eight action labels are used for recognition in this experiment: “Outside the room”, “Transition”, “Seated in a wheelchair”, “Standing”, “Sitting on the bed”, “Lying on the bed”, “Receiving assistance”, and “Falling”. Some example depth images for each label are shown in Figure 14.

As for “Transition” labels, all transition states from one action to another are included. The “Falling” label is not included as an example in Figure 14, as no real falling events were recorded during the experiment. However, this case is defined just in case.

During background subtraction, the “Outside the room” label is used when no object has been detected in the current foreground frame, indicating that no person is detected in the room. Among eight action labels, “Transition”, “Seated in a wheelchair”, “Standing”, “Sitting”, and “Lying down” are used for training SVM by calculating the histograms of DMA and DMH. The outputs from SVM are then checked again against the ROI regions to locate the person in places such as on the floor or the bed. Moreover, the two labels “Receiving assistance” and “Falling” are determined using the height of the person’s silhouette, as well as distance features on the SVM output action labels. The nurse providing assistance is usually taller than the elderly person, and the normal height of an elderly person also appears higher than the one who has fallen on the floor. Thus, two threshold values must be set for this case: if the height of the person is greater than the threshold *Th_assist_*, the action is recognized as “Receiving assistance;” if the height is lower than the threshold *Th_fall_*, the action is recognized as “Falling”. For this reason, the distance feature from the 3D centroid of the person to the floor is extracted in addition to appearance-based features.

#### 2.6.1. Automatic Rounding Method

For the recognition process, short action sequences of various lengths are recognized in the continuous long sequences. For recognition in this experiment, we adopted the idea of using the automatic rounding method [28]. This method automatically divides long frame sequences frame by frame into several short sequences. Deciding whether an action has occurred is a matter of determining the probability of forming an action label from the SVM classifier. If the probability is higher than a pre-determined threshold *Th_prob_*, the round stops, outputs the detected action result, and starts another recognition round for the next sequence.

**Figure 15:**
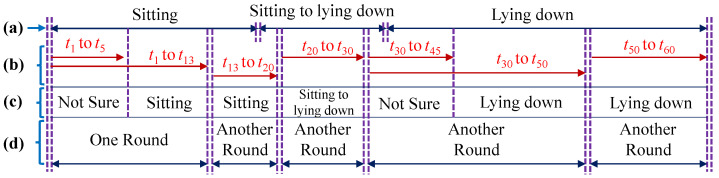
Example of using the automatic rounding method: (**a**) Ground Truth; (**b**) Input Sequence; (**c**) Recognition Process; (**d**) Example rounds.

An example of using the automatic rounding method for a sequence is shown in Figure 15. In this example, three actions occur in the ground truth sequence: “Sitting”, “Sitting to lying down”, and “Lying down”, and the frame rate is 1 fps. When the input sequence is given, the recognition process starts from *t*_1_, and goes frame by frame. The recognizer cannot determine the action until *t*_5_. As time increases, the probability of “Sitting” increases. When it reaches *t*_13_, the probability is higher than the threshold for “Sitting”. The recognizer stops, ends this round, and starts a new recognition round for the next sequence. In this way, short action sequences of various lengths are recognized to the end of a long sequence. If, due to low probability, the recognition round does not stop until reaching a maximum duration (30 s for this experiment), the recognizer considers the sequence to be an undetermined action, and will not label the result.

## 3. Experimental Results

The following provides experimental results and a performance evaluation for the proposed work, using the dataset collected in the elder care center.

### 3.1. Data Preparation

Depth data were collected from three rooms in the elder care center with an image resolution of 320 × 180. The total number of recording days for each room were 9, 6, and 10 days. The total number of sequences identified for each room are 14, 10, and 11, respectively. Each annotated sequence starts when the elderly person entered the room and ends when that person left the room. Table 1 describes all of the sequences in the three rooms with their recording dates and durations. The sequences in the second room were used as training data, and those recorded in the other rooms were used as testing data. The minimum duration for a sequence was one hour during the day, and the maximum was 13 h at night. The elderly person usually enters the room during the day for taking a nap, and at night for sleeping.

### 3.2. Parameter Setting

Table 2 provides the threshold parameters used throughout the process for each section.

### 3.3. Training Process

Person detection and tracking were performed on sequences in the training data, and person silhouettes were first cropped and normalized to a fixed size in the training process. Next, the various action sequences of various lengths were manually annotated in the training sequences. The minimum length of an action sequence for training was five frames (5 s) and the maximum length was 30 frames (30 s). Five action labels were used for a total of 579 sequences, as described in Table 3, which also provides the number of sequences for each label. The DMAs, DMHs, and HOG features for these short action sequences were extracted before being fed to the SVM classifier for training all the sequences.

### 3.4. Testing Process and Performance Evaluation

All of the long sequences recorded in Room 1 and 3 were used for testing with the automatic rounding method, and the results were then evaluated. However, performance was evaluated in both sets of data in random frame sequences. At first, two long sequences were randomly generated from the data for each room, one each using day-time and night-time sequences. The durations of these two long sequences are shown in Table 4.

From each long sequence, three ten-minute sequences were again randomly generated, and they were used for evaluating performance. The accuracy was calculated by comparing the action detected in the same frame in both testing and ground truth sequences. The accuracy for each short sequence is shown in Table 5.

**Figure 16:**
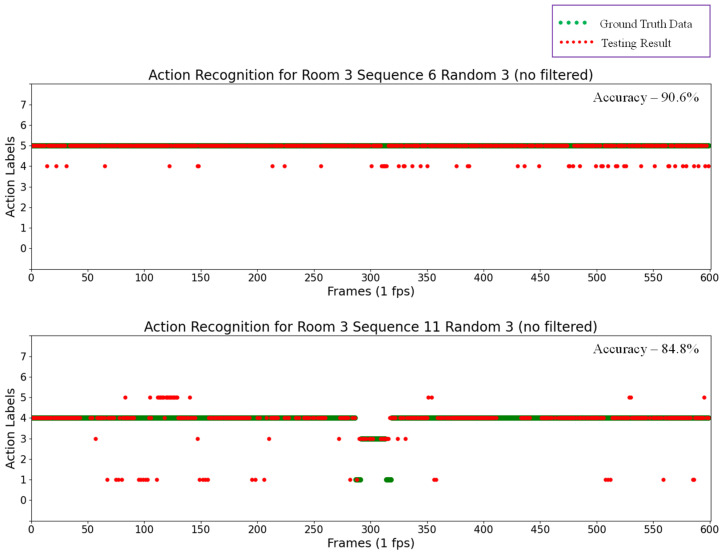
Action recognition results for Room 3 Sequence 6 Random 3 (**top**) and Room 3 Sequence 11 Random 3 (**bottom**).

Labels were numbered in ascending order from 0 to 7 for the eight actions, including “0” for “Outside the room”, “1” for “Transition”, “2” for “Seated in wheelchair”, “3” for “Standing”, “4” for “Sitting on the bed”, ”5” for “Lying on the bed”,, “6” for “Receiving assistance”, and finally, “7” for “Falling”. The results for some random testing sequences are also shown in the scatter plots in Figure 16, in which green points represent ground truth data and red points represent the testing results for action recognition.

**Figure 17:**
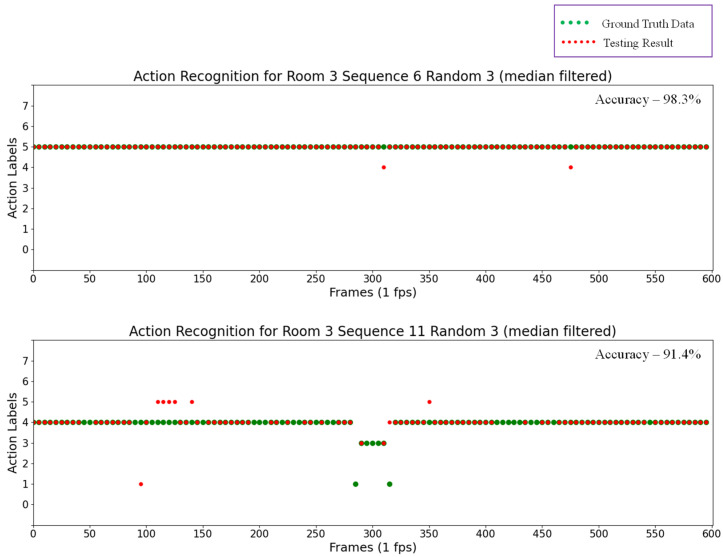
Action recognition results after applying filter for Room 3 Sequence 6 Random 3 (**top**) and Room 3 Sequence 11 Random 3 (**bottom**).

By analyzing the results of these testing sequences, the automatic rounding method can recognize actions in almost every frame with a pre-determined probability, thus determining actions within a short time. However, actions were incorrectly detected in some results. For example, in the upper scatter plot in Figure 16, “Lying on the bed” actions were falsely detected as “Sitting on the bed”, and the number of these false detections was reduced by applying a median filter to the previous action recognition results. The results of thus applying a median filter are shown in Figure 17. In this experiment, the window size for the median filter was five. However, this technique of applying a median filter is time consuming, and not successful in recognizing actions in every frame. For example, in the upper plot in Figure 16, actions can be recognized without the median filter in every frame (600 frames) with an accuracy of 90.6%. After applying the median filter, actions can only be recognized in 120 frames, though with an accuracy of 98.3%. In this technique, the median filter is applied every five frames, and thus it requires five seconds to recognize the action at a time.

Table 6 compares the accuracy for all of the testing sequences. This table shows that the accuracy in some sequences may degrade slightly after using a median filter, but most of the other sequences are more accurate than before. In addition, the lower plot in Figure 17 indicates that the action recognizer cannot detect any action in some frames, showing nothing as a result. As stated in Section 2.6.1, this is due to the recognition process of the rounding method reaching the maximum duration (30 s in this experiment) before the probability becomes sufficient for forming an action. Consequently, the recognizer does not label results for these frames. Furthermore, Table 7, Table 8, Table 9 and Table 10 provide confusion matrices for the results of each random testing sequence after applying a median filter, in which the values indicate the number of detected action frames from the sequences, and diagonal bold numbers represent the number of correctly detected frames for each action.

The testing process was performed using a 64-bit Intel (R) core i7 PC with 32GB RAM (ASUS, Taipei, Taiwan), providing a processing time of about 2/3 of the duration of the continuous long-frame sequences. For instance, the processing time was about 7.7 h for the 12-h duration of the testing sequence in Room 3, Sequence 11. This indicates that our proposed work can be used in a real-time application.

## 4. Discussion and Conclusions

The proposed system in this article focused on a vision-based solution for monitoring and recognizing the various actions of elderly people, using depth maps recorded by a depth camera. This camera ensures the privacy of users and also simplifies plane detection using depth information and UV-disparity maps. We have introduced a system that fuses together feature extraction methods from previous works in a novel combination for action recognition. The experiment tested and evaluated this system using a dataset that was collected at an elder care center. As for feature vectors, appearance-based features [24] (depth motion appearance and depth motion history with HOG descriptor) are used in combination with the distance-based features [21] (3D human centroid height relative to the floor) and fused together with the automatic rounding method [28] for action recognition. The duration that forms a specific action is automatically annotated by the rounding method, and the action is recognized by an SVM classifier according to the feature vector. The spatial-temporal features enabled by a HOG descriptor can also provide rich information in recognizing various actions. Furthermore, the automatic rounding method for long sequences can detect actions in real-time, regardless of the length of the image sequences.

However, our experiment indicates that the proposed action recognition process makes some false detections. These errors usually occur with interaction between person objects and other objects in the foreground, such as blankets, wheelchairs, and the nurse. Furthermore, as depth map features are extracted based on the appearance of the silhouette, false detections happen when occlusion occurs between objects which have distorted silhouette shapes. This problem may be solved by converting the depth maps to 3D point clouds, as well as by clustering and segmentation using the distance information of occluded objects. Therefore, how to improve accuracy during occlusion and interaction between objects will be a subject of further investigation.

To conclude this article, our proposed system will also be relevant in the field of active and assisted living. Through the combination of computer vision with medical technology, this system could enormously improve health quality for the elderly. At the same time, the system could reduce accident rates and the cost of care. On improving accuracy, future research could use improved action histories for analyzing daily patterns of activities and behavior using statistical models such as the hidden Markov model (HMM) [32]. Moreover, in our proposed system, the potential combination of real-time monitoring, fall detection, and further human behavior analysis from action histories offers possibilities for various applications within the field of active and assisted living. These include anomaly detection, sleep quality analysis, and emergency alerting systems. Furthermore, health data can be mined using big data analysis driven by Machine Learning and Artificial Intelligence on a cloud-based data analysis server.

## Figures and Tables

**Table 1:** Long sequences from the dataset.

Room ID	Sequence ID	Data and Time		Duration (h)
		Start	End	
1	1	12 October 2019_20:39	13 October 2019_03:34	7
	2	18 October 2019_12:07	18 October 2019_15:13	3
	3	18 October 2019_17:59	19 October 2019_05:50	12
	4	19 October 2019_08:19	19 October 2019_10:15	2
	5	19 October 2019_18:00	20 October 2019_06:45	13
	6	20 October 2019_12:02	20 October 2019_14:50	3
	7	20 October 2019_19:17	21 October 2019_06:20	11
	8	21 October 2019_12:20	21 October 2019_14:58	3
	9	21 October 2019_19:08	22 October 2019_06:25	12
	10	22 October 2019_11:54	22 October 2019_14:45	3
	11	22 October 2019_18:39	23 October 2019_06:17	12
	12	23 October 2019_12:21	23 October 2019_15:40	3
	13	23 October 2019_17:51	24 October 2019_06:10	12
	14	24 October 2019_12:28	24 October 2019_14:15	2
2	1	21 October 2019_19:03	22 October 2019_06:55	12
	2	22 October 2019_19:53	22 October 2019_20:35	1
	3	25 October 2019_12:18	25 October 2019_15:05	3
	4	25 October 2019_18:24	26 October 2019_06:28	12
	5	26 October 2019_08:09	26 October 2019_09:40	2
	6	26 October 2019_12:11	26 October 2019_14:45	3
	7	26 October 2019_17:44	27 October 2019_06:10	12
	8	27 October 2019_07:52	27 October 2019_10:14	2
	9	27 October 2019_17:44	28 October 2019_06:44	13
	10	28 October 2019_09:16	28 October 2019_10:05	1
3	1	12 October 2019_12:03	12 October 2019_14:50	3
	2	12 October 2019_17:46	13 October 2019_04:40	11
	3	18 October 2019_19:48	19 October 2019_06:30	11
	4	19 October 2019_19:42	20 October 2019_07:42	12
	5	20 October 2019_19:42	21 October 2019_06:35	11
	6	25 October 2019_17:53	26 October 2019_06:30	12
	7	26 October 2019_08:11	26 October 2019_09:00	1
	8	26 October 2019_18:00	27 October 2019_06:08	12
	9	27 October 2019_08:08	27 October 2019_09:42	2
	10	27 October 2019_18:10	28 October 2019_06:46	12
	11	28 October 2019_08:12	28 October 2019_10:23	2

**Table 2:** Threshold parameter values used throughout the process.

Sections	Threshold Parameters	Values
Background Subtraction	*Th_bg_sub_*	0.2*σ* ^1^
Object Detection and Tracking	*Th_motion_*	80
Object Detection and Tracking	*Th_dist_*	30
Object Detection and Tracking	*Th_area_*	5000
Object Detection and Tracking	*Th_depth_*	0.5
Feature Extraction (Appearance-based)	*δ*	0.01
Feature Extraction (Distance-based)	*Th_assist_*	1.0
Feature Extraction (Distance-based)	*Th_fall_*	0.4
Action Recognition	*Th_prob_*	30

^1^*σ* depends on the standard deviation of input depth image.

**Table 3:** Number of training sequences for five action labels.

Action Labels	Training Sequences
Transition	65
Seated in Wheelchair	80
Standing	91
Sitting	243
Lying	100

**Table 4:** Randomly generated long sequences for testing data.

Room ID	Sequence ID	Data and Time		Duration (h)
		Start	End	
1	7	20 October 2019_19:17	21 October 2019_06:20	11
	8	21 October 2019_12:20	21 October 2019_14:58	3
3	6	25 October 2019_17:53	26 October 2019_06:30	12
	11	28 October 2019_08:12	28 October 2019_10:23	2

**Table 5:** Accuracy for the randomly generated testing sequences.

Room ID	Sequence ID	Random ID	Total Frames	Duration (min)	Accuracy (%)
1	7	1	600	10	100
		2	600	10	95.6
		3	600	10	100
1	8	1	600	10	69.2
		2	600	10	100
		3	600	10	100
3	6	1	600	10	86.6
		2	600	10	94.7
		3	600	10	90.6
3	11	1	600	10	88.8
		2	600	10	83.9
		3	600	10	84.8

**Table 6:** Comparison of accuracy for the randomly generated testing sequences.

Room ID	Sequence ID	Random ID	Total Frames	Duration (min)	Accuracy (%) (without Filter)	Accuracy (%) (with Filter)
1	7	1	600	10	100	100
		2	600	10	95.6	94.6
		3	600	10	100	100
1	8	1	600	10	69.2	70.9
		2	600	10	100	100
		3	600	10	100	100
3	6	1	600	10	86.6	94.2
		2	600	10	94.7	100
		3	600	10	90.6	98.3
3	11	1	600	10	88.8	86.8
		2	600	10	83.9	88.5
		3	600	10	84.8	91.4

**Table 7:** Confusion matrix for Room 1 Sequence 7 (with median filter).

		Predict																							
		Random-1 (Accuracy: 100%)								Random-2 (Accuracy: 94.6%)								Random-3 (Accuracy: 100%)							
		**0**	**1**	**2**	**3**	**4**	**5**	**6**	**7**	**0**	**1**	**2**	**3**	**4**	**5**	**6**	**7**	**0**	**1**	**2**	**3**	**4**	**5**	**6**	**7**
**Actual**	**0**	**0**	0	0	0	0	0	0	0	**0**	0	0	0	0	0	0	0	**0**	0	0	0	0	0	0	0
	**1**	0	**0**	0	0	0	0	0	0	0	**0**	0	0	0	0	0	0	0	**0**	0	0	0	0	0	0
	**2**	0	0	**0**	0	0	0	0	0	0	0	**0**	0	0	0	0	0	0	0	**0**	0	0	0	0	0
	**3**	0	0	0	**0**	0	0	0	0	0	0	0	**0**	0	0	0	0	0	0	0	**0**	0	0	0	0
	**4**	0	0	0	0	**0**	0	0	0	0	0	0	0	**24**	6	0	0	0	0	0	0	**0**	0	0	0
	**5**	0	0	0	0	0	**120**	0	0	0	0	0	0	0	**82**	0	0	0	0	0	0	0	**120**	0	0
	**6**	0	0	0	0	0	0	**0**	0	0	0	0	0	0	0	**0**	0	0	0	0	0	0	0	**0**	0
	**7**	0	0	0	0	0	0	0	**0**	0	0	0	0	0	0	0	**0**	0	0	0	0	0	0	0	**0**

**Table 8:** Confusion matrix for Room 1 Sequence 8 (with median filter).

		Predict																							
		Random-1 (Accuracy: 70.9%)								Random-2 (Accuracy: 100%)								Random-3 (Accuracy: 100%)							
		**0**	**1**	**2**	**3**	**4**	**5**	**6**	**7**	**0**	**1**	**2**	**3**	**4**	**5**	**6**	**7**	**0**	**1**	**2**	**3**	**4**	**5**	**6**	**7**
**Actual**	**0**	**10**	0	0	0	0	0	0	0	**0**	0	0	0	0	0	0	0	**0**	0	0	0	0	0	0	0
	**1**	0	**0**	3	0	2	2	0	0	0	**0**	0	0	0	0	0	0	0	**0**	0	0	0	0	0	0
	**2**	0	0	**0**	0	0	0	0	0	0	0	**0**	0	0	0	0	0	0	0	**0**	0	0	0	0	0
	**3**	0	0	0	**0**	0	0	0	0	0	0	0	**0**	0	0	0	0	0	0	0	**0**	0	0	0	0
	**4**	0	0	2	3	**28**	16	1	0	0	0	0	0	**0**	0	0	0	0	0	0	0	**0**	0	0	0
	**5**	0	0	0	0	0	**36**	0	0	0	0	0	0	0	**120**	0	0	0	0	0	0	0	**120**	0	0
	**6**	0	0	1	1	0	1	**4**	0	0	0	0	0	0	0	**0**	0	0	0	0	0	0	0	**0**	0
	**7**	0	0	0	0	0	0	0	**0**	0	0	0	0	0	0	0	**0**	0	0	0	0	0	0	0	**0**

**Table 9:** Confusion matrix for Room 3 Sequence 6 (with median filter).

		Predict																							
		Random-1 (Accuracy: 94.2%)								Random-2 (Accuracy: 100%)								Random-3 (Accuracy: 98.3%)							
		**0**	**1**	**2**	**3**	**4**	**5**	**6**	**7**	**0**	**1**	**2**	**3**	**4**	**5**	**6**	**7**	**0**	**1**	**2**	**3**	**4**	**5**	**6**	**7**
**Actual**	**0**	**0**	0	0	0	0	0	0	0	**0**	0	0	0	0	0	0	0	**0**	0	0	0	0	0	0	0
	**1**	0	**0**	0	0	1	0	0	0	0	**0**	0	0	0	0	0	0	0	**0**	0	0	0	0	0	0
	**2**	0	0	**0**	0	0	0	0	0	0	0	**0**	0	0	0	0	0	0	0	**0**	0	0	0	0	0
	**3**	0	0	0	**6**	3	0	0	0	0	0	0	**0**	0	0	0	0	0	0	0	**0**	0	0	0	0
	**4**	0	0	0	0	**59**	0	0	0	0	0	0	0	**34**	0	0	0	0	0	0	0	**0**	0	0	0
	**5**	0	0	0	0	0	**0**	0	0	0	0	0	0	0	**0**	0	0	0	0	0	0	2	**118**	0	0
	**6**	0	0	0	0	0	0	**0**	0	0	0	0	0	0	0	**0**	0	0	0	0	0	0	0	**0**	0
	**7**	0	0	0	0	0	0	0	**0**	0	0	0	0	0	0	0	**0**	0	0	0	0	0	0	0	**0**

**Table 10:** Confusion matrix for Room 3 Sequence 11 (with median filter).

		Predict																							
		Random-1 (Accuracy: 86.8%)								Random-2 (Accuracy: 88.5%)								Random-3 (Accuracy: 91.4%)							
		**0**	**1**	**2**	**3**	**4**	**5**	**6**	**7**	**0**	**1**	**2**	**3**	**4**	**5**	**6**	**7**	**0**	**1**	**2**	**3**	**4**	**5**	**6**	**7**
**Actual**	**0**	**0**	0	0	0	0	0	0	0	**23**	0	0	0	0	0	0	0	**0**	0	0	0	0	0	0	0
	**1**	0	**0**	0	0	3	0	0	0	0	**0**	1	0	1	0	0	0	0	**0**	0	0	1	0	0	0
	**2**	0	0	**0**	0	0	0	0	0	0	0	**14**	0	0	0	0	0	0	0	**0**	0	0	0	0	0
	**3**	0	0	0	**0**	3	0	0	0	0	0	0	**3**	0	0	0	0	0	0	0	**2**	0	0	0	0
	**4**	0	0	0	0	**79**	6	0	0	0	0	1	2	**14**	2	0	0	0	1	0	0	**83**	6	0	0
	**5**	0	0	0	0	0	**0**	0	0	0	0	0	0	0	**0**	0	0	0	0	0	0	0	**0**	0	0
	**6**	0	0	0	0	0	0	**0**	0	0	0	0	0	0	0	**0**	0	0	0	0	0	0	0	**0**	0
	**7**	0	0	0	0	0	0	0	**0**	0	0	0	0	0	0	0	**0**	0	0	0	0	0	0	0	**0**

## Data Availability

The data presented in this study are available on request from the corresponding author.
